# High Throughput Chemical Screening Reveals Multiple Regulatory Proteins on FOXA1 in Breast Cancer Cell Lines

**DOI:** 10.3390/ijms19124123

**Published:** 2018-12-19

**Authors:** Shixiong Wang, Sachin Kumar Singh, Madhumohan R. Katika, Sandra Lopez-Aviles, Antoni Hurtado

**Affiliations:** 1 Breast Cancer Research group, Nordic EMBL Partnership, Centre for Molecular Medicine Norway (NCMM), University of Oslo, P.O. 1137 Blindern, 0318 Oslo, Norway; parmarsachinsingh@gmail.com (S.K.S.); maddycdfd@gmail.com (M.R.K.); 2 Cell Cycle Research group, Nordic EMBL Partnership, Centre for Molecular Medicine Norway (NCMM), University of Oslo, P.O. 1137 Blindern, 0318 Oslo, Norway; s.l.aviles@ncmm.uio.no

**Keywords:** breast cancer, FOXA1, drug screening and proteomics

## Abstract

Forkhead box A1 (FOXA1) belongs to the forkhead class transcription factor family, playing pioneering function for hormone receptors in breast and prostate cancers, and mediating activation of linage specific enhancers. Interplay between FOXA1 and breast cancer specific signaling pathways has been reported previously, indicating a regulation network on FOXA1 in breast cancer cells. Here in this study, we aimed to identify which are the proteins that could potentially control FOXA1 function in breast cancer cell lines expressing different molecular markers. We first established a luciferase reporter system reflecting FOXA1 binding to DNA. Then, we applied high throughput chemical screening of multiple protein targets and mass spectrometry in breast cancer cell lines expressing different molecular markers: ER positive/HER2 negative (MCF-7), ER positive/HER2 positive (BT474), and ER negative/HER2 positive (MDA-MB-453). Regardless of estrogen receptor status, HER2 (human epidermal growth factor receptor 2) enriched cell lines showed similar response to kinase inhibitors, indicating the control of FOXA1 by cell signaling kinases. Among these kinases, we identified additional receptor tyrosine kinases and cyclin-dependent kinases as regulators of FOXA1. Furthermore, we performed proteomics experiments from FOXA1 inmunoprecipitated protein complex to identify that FOXA1 interacts with several proteins. Among all the targets, we identified cyclin-dependent kinase 1 (CDK1) as a positive factor to interact with FOXA1 in BT474 cell line. In silico analyses confirmed that cyclin-dependent kinases might be the kinases responsible for FOXA1 phosphorylation at the Forkhead domain and the transactivation domain. These results reveal that FOXA1 is potentially regulated by multiple kinases. The cell cycle control kinase CDK1 might control directly FOXA1 by phosphorylation and other kinases indirectly by means of regulating other proteins.

## 1. Introduction

Forkhead Box A1 (FOXA1), also known as hepatocyte nuclear factor 3 alpha, belongs to the forkhead family of transcription factors and plays pivotal roles in the development of prostate and mammary gland [1,2]. FOXA1 is a pioneer transcription factor with a DNA binding domain that resembles the structure of linker histones [3,4]. Moreover, the C-terminal domain interacts with the core histones to endorse chromatin opening [5]. Genome-wide studies have shown that FOXA1 is enriched at regions with enhancer histone marks H3K4me1 and H3K4me2 [6] and that mediates the activation of lineage enhancers [7]. Recently, it has been reported that FOXA1 recruits histone methyl transferase to mediate the methylation on H3K4me1 [8]. FOXA1 is also able to control the expression of TET1 and interact with it to regulate local DNA methylation and the activity of corresponding enhancer activity [9]. These properties confer the ability of FOXA1 to act as a pioneer factor for both estrogen receptor α [10,11] and androgen receptor [12], controlling their binding, location, and function in breast and prostate cancer respectively.

Breast cancer is the most common cancer type among women in developed countries. Breast cancer is a heterogeneous disease with several subtypes showing differences in histopathology, tumor biology, and prognosis. Despite of such complexity, around 70% of breast cancers cases are estrogen receptor alpha (ER) positive. ER is a nuclear receptor that mediates the response to estrogen and triggers a transcription program driving the proliferation of cancer cells [13,14]. Therefore, ER and its associated metabolism have been the targets of endocrine therapies, which have significantly improved the survival rate of ER positive breast cancer patients [15]. However, resistance to endocrine therapies has been observed in a substantial fraction of ER positive patients. The resistance is often associated with gain of receptor tyrosine kinase function [15] such as ERBB2/HER2. In addition, ER somatic mutations can also result in hormone resistance by activating the receptor in the absence of ligand binding in metastatic ER positive tumors [16,17]. Interestingly, almost all the ER binding chromatin interactions are dependent on the pioneer factor FOXA1, a dependence that is even preserved in hormone resistant tumors [10]. ER and FOXA1 are co-expressed in metastatic endocrine resistant tumor and the redistribution of ER binding correlates with FOXA1 binding [18]. Moreover, overexpression of FOXA1 also mediates endocrine resistance by varying the ER-regulated transcripts and the IL-8 signaling in preclinical model [19]. These particular properties support the idea that FOXA1 could be an attractive target of ER positive breast cancer especially in endocrine resistant context. However, targeting directly a pioneer factor might provoke undesired side effects in healthy tissues. On the other hand, targeting proteins with a role activating FOXA1 could be a plausible alternative. Hence, in this study, we aimed to investigate which proteins can control FOXA1 in different breast cancer cell lines. For this aim, we took a high throughput chemical screening approach (with known protein targets) in order to search for proteins controlling FOXA1 in breast cancer cells. As readout, we used a luciferase reporter system, which is able to reflect FOXA1 binding to DNA. After two rounds of high throughput chemical screening, we identified several interesting proteins that could be potential FOXA1 regulators. Finally, by means of performing proteomics experiments we could identify that cyclin-dependent kinases 1 (CDK1) might directly regulate FOXA1 by phosphorylation.

## 2. Results

### 2.1. Generation of the FOXA1 Luciferase Reporter System

**Figure 1:**
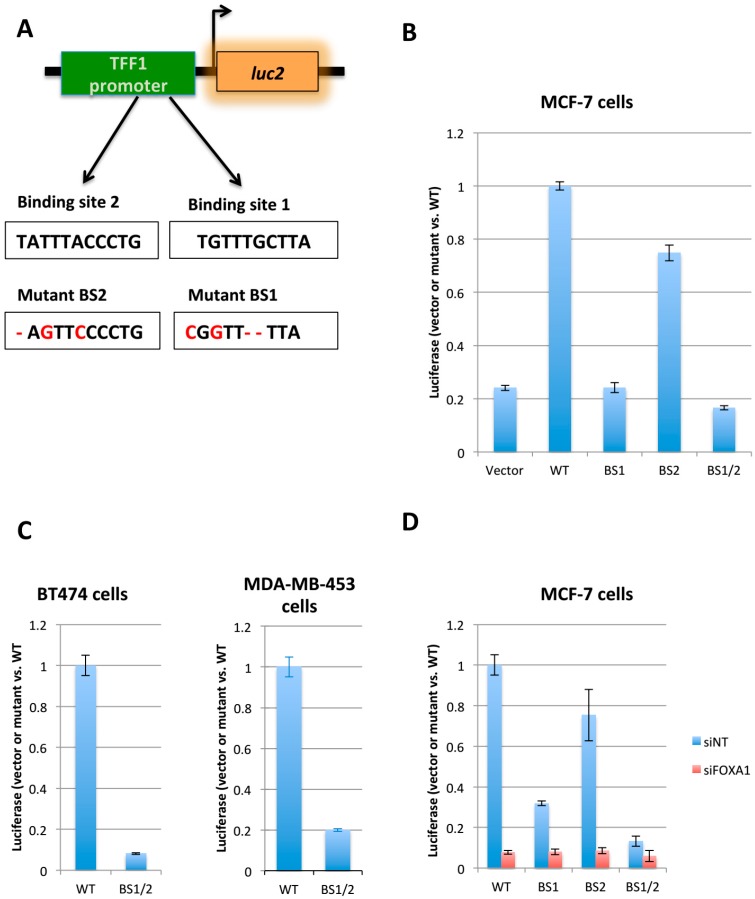
Design of the luciferase reporter and validation. (**A**) Schematic design of the luciferase reporter reflecting FOXA1 activity and the mutations of FOXA1 binding sites. The promoter of Tff1 gene was cloned into the upstream of the luciferase expression cassette of pGL4.20 vector, two FOXA1 motifs are mutated to the sequences in red. (**B**) The wild type reporter (WT), reporter with the mutation in BS1 (BS1), BS2 (BS2), and double mutant (BS1/2) were transfected into MCF-7 cells for validation. Luciferase assay was performed to measure the reporter activity (*n* = 3). (**C**) Wild type and double mutant reporter plasmids were validated further with BT474 (**left**) and MDA-MB-453 (**right**) cell lines (*n* = 3). (**D**) The pGL4.20-WT, BS1, BS2, and BS1/2 were transfected into MCF-7 together with non-targeting siRNA (siNT) and siRNA targeting FOXA1 (siFOXA1). Luciferase assay was performed 48 h after transfection (*n* = 3).

In order to perform the high throughput screening, we constructed a luciferase reporter system reflecting the binding of FOXA1 to forkhead motifs. The promoter of the TFF1 gene, which contains two forkhead motifs, was cloned upstream of the luciferase expression cassette of pGL4.20 plasmid (Figure 1A). Previously, it was demonstrated that with a similar construct the luciferase expression was controlled by the FOXA1 binding to the promoter of the chosen gene [20]. To validate the system, we transfected the reporter plasmid into MCF-7 (ER positive/HER2 negative), BT474 (ER positive/HER2 positive), and MDA-MB-453 (ER negative/HER2 positive) breast cancer cell lines. We choose MCF-7 and BT474 cell lines is because they are positive for the expression of ER. The cell line MDA-MB-453 here serves as an ER negative control cell line. The luciferase assay was performed 48 h after transfection. In MCF-7, the reporter showed a much higher activity than the empty vector control, which means that the promoter can drive the expression of the downstream luciferase reporter (Figure 1B). To further validate that the luciferase signal was FOXA1 dependent, the two-forkhead motifs in the promoter were mutated. Hence, the corresponding created mutants were named BS1 (binding site 1 mutated), BS2 (binding site 2 mutated) and BS1/2 (both 1 and 2 mutated). In MCF-7, the mutation of BS1 almost abolished the reporter activity to the level of the empty vector control, while the BS2 only reduced the signal around 20% (Figure 1B). The data suggested that the BS1 site played the main role in mediating FOXA1 binding. The double mutant BS1/2 also showed a significant reduction of luciferase signal in all cell lines tested (Figure 1B,C), with a level similar to BS1 in MCF-7. Moreover, knockdown of FOXA1 with siRNA in MCF-7 also abolished the activity of the WT reporter (Figure 1D), which confirmed the FOXA1 specificity of the reporter system. Previously, it has been reported that both ER and FOXA1 bind to TTF1 promoter and induce its expression [20]. Importantly, our experiments were performed in estrogen-depleted conditions, which is a condition that impedes the binding of ER to the TFF1 promoter. In Figure S1 the binding of ER and FOXA1 at the promoter of TFF1-LUC construct is shown. We performed chromatin immunoprecipitation (ChIP) of both transcription factors in MCF7 cells transfected with the TFF1-LUC-WT and TFF1-LUC-mutant (BS1/2) in estrogen-depleted cells followed by real-time PCR. The experiment reveals that FOXA1 interacts to the promoter of TFF1-LUC construct but ER interaction is almost undetectable. Moreover, the data shown in Figure S1 demonstrate that FOXA1 binds to the promoter of TFF1-LUC-WT vector and that the binding disappears in cells carrying mutations of the FKH motif (BS1/2). Hence, our data reveals clearly that the luciferase signal is mainly mostly due to FOXA1 binding.

### 2.2. Multiple Targets Were Identified as Potential FOXA1 Regulators

To test the hypothesis that FOXA1 could be regulated by multiple kinases/proteins, we performed a high throughput chemical screening with the reporter system constructed above. The screening pipeline is illustrated in Figure S2. Briefly, the luciferase reporter was transfected into all MCF-7, BT474, and MDA-MB-453 breast cancer cell lines overnight. Then, cells were re-plated into 384 well plates and maintained in DMEM media free of hormones overnight. Cells were treated with chemicals from a drug library (Enzo Life Sciences; http://www.enzolifesciences.com/) at 10μM concentration. A total of 550 drugs (Table S1) were used in the screening and the luciferase assay was performed 24 h after the start of chemical treatment.

**Figure 2:**
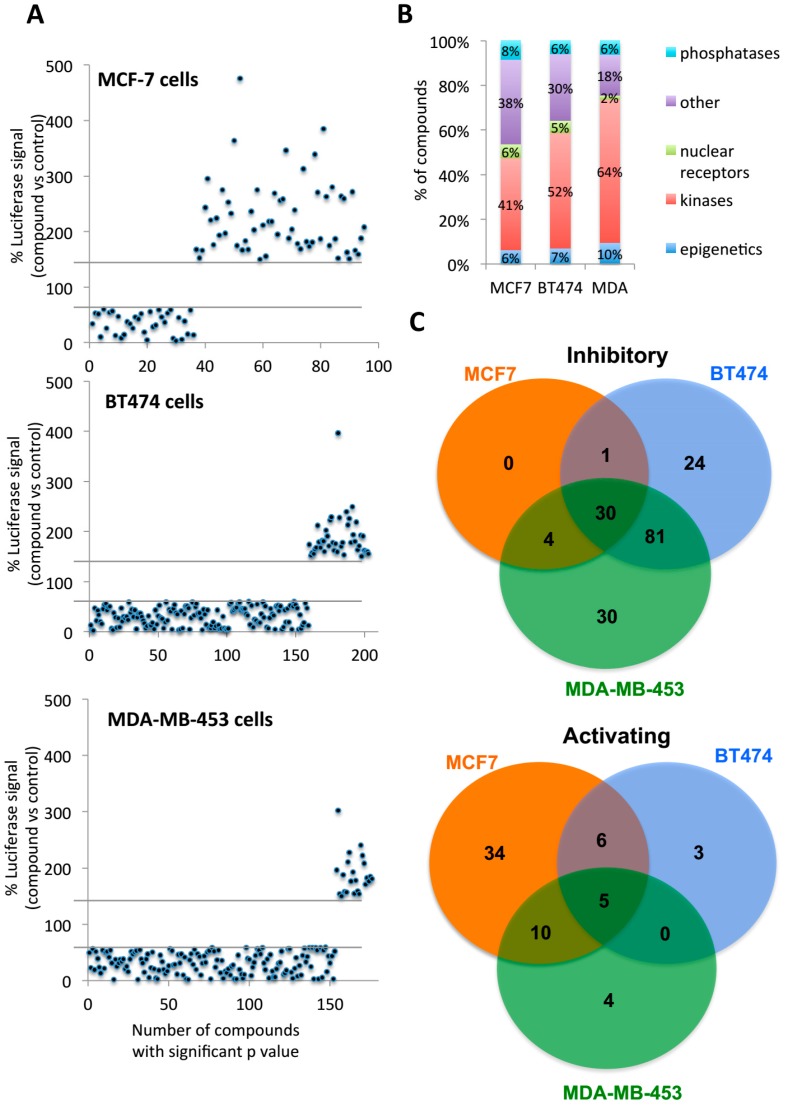
Multiple chemicals were identified positive in the chemical screening. (**A**) Three plots indicating the number of significant compounds (*T* test; two tails; *p* < 0.05) that affect the luciferase expression in each of the breast cancer cell lines investigated (MCF-7, BT474, and MDA-MB-453). Each plot illustrates the % of luciferase expression of cells treated with compounds and normalized to control treated cells (treatment/control). We have represented the compounds with a significant increase (more than 150%) or decrease (less than 40%) luciferase expression compared to control. (**B**) Fraction (expressed in %) of significant compounds targeting different group of proteins: phosphatases, nuclear receptors, kinases, epigenetics and other groups. The plot represents the % of group of compounds with a significant p value for each cell line investigated. (**C**) Venn-diagram showing the overlap of positive chemicals between MCF-7, BT474, and MDA-MB-453 cells. Inhibitory (**upper**) and activating (**lower**) are showed independently. The number of positive chemicals in MCF-7, BT474, and MDA-MB-453 were showed in different columns with activating chemicals in red and inhibitory chemicals in blue.

The data from the chemical screening was analyzed, and drugs with a significant impact were selected based on the fold change of the luciferase signal (T test comparing control treated vs. treated with drug; *p*-value < 0.05 was used as cut-off). We considered uniquely drugs that resulted in an increase of at least 50% or in a reduction of at least 40% of the luciferase signal. Based on the idea that luciferase signal correlates with FOXA1 binding to the TTF1 promoter, the compounds that provide a gain of luciferase signal might be targeting proteins that inhibit FOXA1 binding to the chromatin. By contrast, those compounds that provide a loss of luciferase signal might be targeting proteins that stimulate FOXA1 binding to the chromatin. The numbers of total drugs with a significant impact on luciferase signal from each cell line are summarized in Figure 2A. In MCF-7 cells (HER2 negative), more chemicals that increased luciferase signal were identified compared to inhibitory chemicals (55 and 35, respectively). BT474 cells (HER2 positive) showed a different behavior with fewer chemicals increasing the luciferase signal compared to inhibitory chemicals (14 and 136, respectively). Moreover, the analysis of the other HER2 positive cell line (MDA-MB-453) revealed similar results as the ones obtained with BT474 cells (19 and 144 chemicals that increased or decreased luciferase signal, respectively). Next, we evaluated which were the proteins targeted by the chemicals identified in our screening and which group of proteins they were enriched for each of the cell lines investigated. Our results revealed that the highest fraction of the compounds were kinase inhibitors (Figure 2B). Interestingly, the fraction of compounds was greater in HER2 positive cells (52% in BT474 and 64% in MDA-MB-453) compared to HER2 negative (41% in MCF-7). This finding correlates with the activity of signaling pathways (kinases) in the corresponding cell lines. Due to the high HER2 level in BT474 and MDA-MB-453, cellular signaling pathways in these two cell lines are highly active. By contrast, MCF-7 cell growth is mainly dependent on ER induced transcription, and signaling pathways are not as active as in HER2 positive cell lines. Hence, our data suggests that additional active kinases in signaling pathways may contribute to FOXA1 activity in cell lines with high HER2. Interestingly when we analyzed the overlap between the different cell lines we observed that BT474 and MDA-MB-453 shared most of their inhibitory chemicals (Figure 2C). Altogether, these results indicate a high similarity of kinases that potentially regulate FOXA1 in these two HER2 positive cell lines regardless of ER status.

### 2.3. Second Screening Narrowed down the Number of Compound Target Candidates

**Figure 3:**
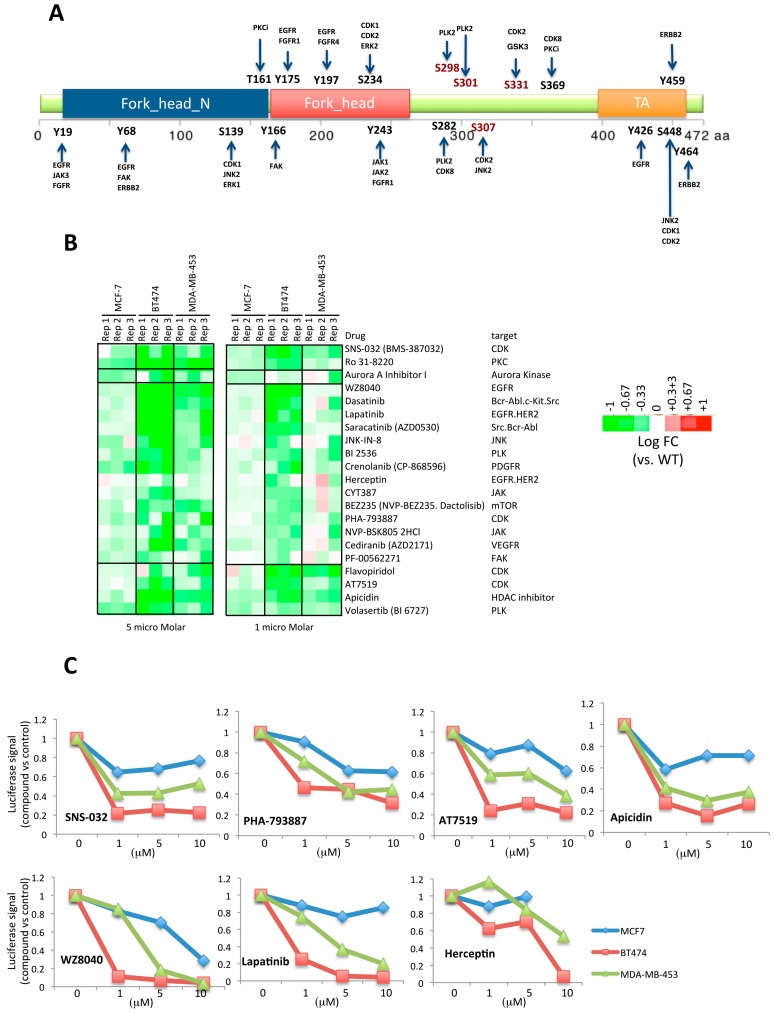
Validation of chemicals by the second screening. (**A**) Diagram showing potential FOXA1 phosphorylation sites and corresponding kinases. The prediction of FOXA1 phosphorylation sites was performed with GPS 3.0, and corresponding kinases that overlapped with positive targets in the first chemical screening were identified. Both overlapping kinases and their potential sites are labeled. (**B**) Heatmap showing the result of compounds with a significant change in luciferase expression. 45 chemicals were used for the second chemical screening for three cell lines and two concentrations (5 and 1 microM). The heatmap illustrates the log2 fold change in luciferase expression (drug treatment vs. control) of cells treated with the 21 compounds with a significant p value. (**C**) Dose responses of compounds with a significant inhibitory effect in the expression of luciferase. The plots represent the relative signal of the compounds targeting the receptor tyrosine kinases HER2/EGFR and the cyclin-dependent kinases (CDK) vs. control treated cells. The average of three independent experiments for different concentrations tested (0, 1, 5, and 10 micro Molar) is plotted.

In order to increase the specificity of the screening and narrow down the number of positive drugs (and their respective targets) for functional validation, a second round of chemical screening was performed using fewer chemicals and lower concentrations. We were more interested in targets that activate FOXA1 and thus only inhibitory drugs from the first screening were selected. In addition, considering that most of the inhibitory chemicals were kinase inhibitors, we performed an in silico phosphorylation prediction using Group-based Prediction System 3.0 (GPS 3.0) [21], in order to identify potential phosphorylation sites in FOXA1. The result of the analysis showed that multiple sites in FOXA1 are potential phosphorylation sites for different kinases. By comparing the in silico phosphorylation analysis and the targets of positive chemicals from the screening (Figure 3A), a list of 45 chemicals were selected for the second round of screening at 5 and 1 μM concentrations using MCF-7, BT474, and MDA-MB-453 cell lines.

The results with the lower concentration used (1 μM) revealed that 21 chemicals were still positive as inhibitors of the reporter. Moreover, some of these compounds were cell type specific (Figure 3B,C, Figures S3 and S4). The results of the screening have identified interesting targets, some of them that have already been shown to be associated with hormone resistance. In this regard, we have identified two inhibitors targeting CDK and PKC in all cell lines. A few chemicals were still shared by BT474 and MDA-MB-453 (4 chemicals), while more than half of all the chemicals (14 of 21 chemicals) were mostly targeting proteins in BT474 cells. These chemicals targeted CDK, receptor tyrosine kinases (EGFR, VEGFR, PDGFR), PLK, JAK, and other intracellular kinases such as PKC, JNK. Finally, an Aurora kinase inhibitor was mainly identified as a positive drug in MCF-7 cells.

### 2.4. FOXA1 Pulldown and Proteomics Identify CDK1 as a Potential Direct Regulator

**Figure 4:**
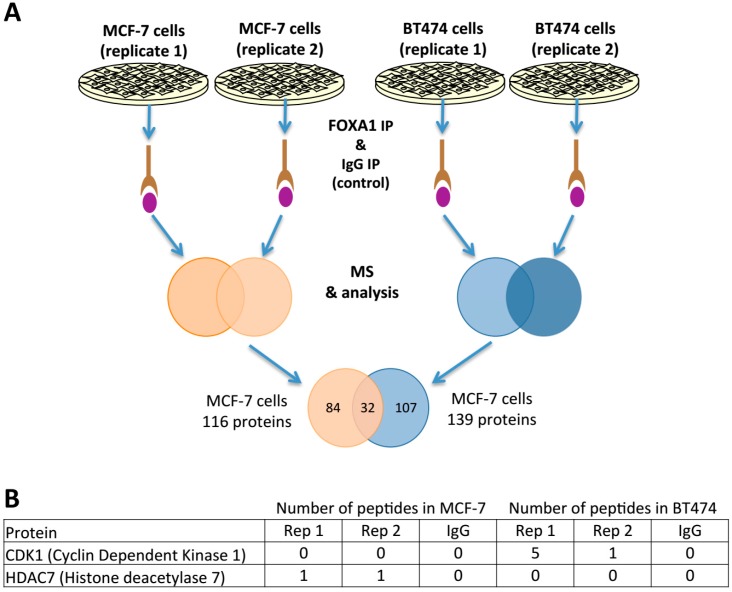
Proteomics results from FOXA1 immunoprecipitation (IP). (**A**) Workflow for FOXA1 IP proteomics. Two independent replicates were performed and uniquely proteins without peptides identified at IgG control were considered positive. Moreover, we considered exclusively proteins identified in both of the replicates. The figure includes a Venn diagram that compares the number of FOXA1 interacting proteins shared between MCF-7 and BT474 cells and the ones identified exclusively each cell line tested. (**B**) Peptide enrichment of CDK1 and HDAC7 in both cell lines is included. The rest of peptide enrichment of the other identified proteins can be found at supplementary information.

Taken together, data above showed that several kinases might control positively FOXA1 function. Moreover, our data suggests that more kinases impinge on FOXA1 function/activity in HER2 positive cell lines. Next, we aimed to identify whether any of the kinases identified from our drug screening might be directly regulating FOXA1. Hence, we used proteomic approaches to identify protein targets of FOXA1 for breast cancer cells. For that, we performed mass spectrometry in MCF-7 and BT474 cells from chromatin immunoprecipitation extracts by using a specific FOXA1 antibody. This resulted in 116 and 139 proteins identified to be interacting with FOXA1 in MCF-7 and BT474 cells, respectively (Table S2). Importantly, this proteomic approach allowed us to identify that around 28% (32 proteins) of the FOXA1 pulled down proteins in MCF-7 were also identified within the FOXA1 pull down in BT474, which confirms the suitability of this method to identify FOXA1-interacting proteins (Figure 4A). In addition, we have also identified other FOXA1 proteins likely to be specific for each cell type investigated. Among these targets identified in BT474 in the chemical screening, we have found CDK1 as a FOXA1 protein partner in the proteomics experiment (Figure 4B).

## 3. Discussion

This work has revealed that several drugs targeting kinases influence the binding of FOXA1 to our TTF1-LUC reporter cassette. Interestingly, the findings from the drug screening indicate that additional kinases have a positive control in HER2 positive cells compared to MCF-7 cells. These results suggest the hypothesis that the binding of FOXA1 to the chromatin in HER2 enriched cells is induced by additional kinases compared to HER2 negative cell lines. The increased number of kinases that positively regulate FOXA1 in HER2 positive cells might be associated with a gain of binding to chromatin. Another possible interpretation of that finding might be understood as a mechanism by which HER2 positive cells guarantee the FOXA1 binding to the chromatin by means of increasing the number of kinases that positively regulate FOXA1. In this regard, it has been recently reported that PI3K might influence the binding of FOXA1 to the chromatin by regulating the activity of the methyltransferase enzyme MLL2 [22]. These enzymes can directly methylate histone H3 on position 4, which are in fact the epigenetic mark recognized by FOXA1 in order to interact with chromatin. Hence, the inhibition of MLL2 by PI3K impacts negatively in the binding of FOXA1 to the chromatin. These results might be in contradiction to our findings, which suggest that the binding of FOXA1 to the DNA might be positively regulated by the same kinase. One possibility of such discrepancies might be due to PI3K inhibiting the subset of FOXA1 chromatin binding regions enriched with DNA sequence motifs for ER interaction. Such hypothesis is supported by the fact that the treatment of PI3K increases the sensitivity of breast cancer cells to anti-estrogen drugs. Hence, the inhibition of PI3K might facilitate indirectly the binding of ER by allowing the binding of FOXA1 and therefore the pioneer function of FOXA1 to ER enriched regions as it has been already reported [23]. Moreover, these results are not in disagreement with the fact that the binding of FOXA1 to additional chromatin regions might be also induced by PI3K/mTOR kinase. In this regard, our drug screening data suggest that mTOR might impact FOXA1 indirectly by other mechanisms that do not imply FOXA1 phosphorylation. In this regard, in our drug screening we have identified that mTOR inhibitors have a negative impact in the binding of FOXA1 to chromatin. Interestingly, it has been reported that the pharmacologic inhibition of GSK3 antagonizes the suppressive effects on the growth of mTOR inhibitors [24,25], which suggests that the kinase GSK3 might be operating downstream of mTOR. Given these evidences, together with the results of this study showing that Ser 331 of FOXA1 contains a consensus site for GSK3 phosphorylation, we might hypothesize that mTOR inhibition affects FOXA1 binding to DNA through the regulation of GSK3. Whether this phosphorylation confers an increase of activity of FOXA1 needs to be elucidated yet.

In this study, we have observed that CDK1 is a protein that interacts with FOXA1 in HER2 positive cells. The interplay between FOXA1 and CDK1 has not being investigated previously. In this regard, it has been reported that CDK1 can regulate gene transcription at S phase of the cell cycle [26]. In particular, CDK1 phosphorylates key transcription factors in S phase and regulates their activity and protein stability [26]. Moreover, a previous study [27] has reported that FOXA1 binds to chromatin in mitosis. This study has also reported that the FOXA1 mitotic binding helps cells to prepare for the transcriptional reactivation of interphase genes after mitotic exit. Importantly, CDK1 activity increases during mitosis, leading to the phosphorylation of proteins whose function is required during this phase of the cell cycle. At the metaphase to anaphase transition, degradation of cyclin B results in a drop in CDK1 activity and mitotic exit. Considering that FOXA1 interacts with CDK1 in BT474 cells but not in MCF-7 cells, one might hypothesize that FOXA1 might be regulated by CDK1 to prepare cells for the transcriptional reactivation of genes just after mitosis. Our in silico analyses for putative sites of phosphorylation have identified several CDK consensus motifs in FOXA1. Moreover, we have observed that the treatment with several general CDK inhibitors have a negative impact on FOXA1 binding to chromatin. Interestingly, this effect was stronger in BT474 compared to the other cell lines investigated. Altogether it is reasonable to postulate that FOXA1 is a potential substrate of CDK1, and the mitotic chromatin binding of FOXA1 could be regulated by CDK1 phosphorylation. If the hypothesis is correct, inhibitors repressing CDK activity should influence the affinity between FOXA1 and its target binding motifs, as observed for the TFF1 reporter. Future experiments might confirm whether FOXA1 is a substrate of CDK1 and should also resolve how such phosphorylation impacts its function.

## 4. Materials and Methods

### 4.1. Cell Culture

MCF-7, BT-474, and MDA-MB-453 cell lines were purchased from American Type Culture Collection (ATCC, Manassas, VA, USA). MCF-7 and MDA-MB-453 cell lines were cultured in in DMEM (4.5 g/L glucose) supplemented with 10% FBS BT474 was cultured in DMEM (4.5 g/L glucose) supplemented with 10% FBS, and 0.01 mg/mL insulin.

### 4.2. Construction of the Luciferase Reporter System

pGL4.20 plasmid was purchased from Promega (E6751). The sequence of wild type TFF1 promoter was obtained from UCSC genome browser (http://genome.ucsc.edu/) with the genomic coordinate chr21:43,786,510-43,787,509 of the GRCh37/hg19 assembly. The promoter of TFF1 gene was cloned into the pGL4.20 with Mul I and Bgl II restriction sites. FOXA1 binding sites mutagenesis was performed with QuikChange II Site-Directed Mutagenesis Kit (200524, Agilent, Santa Clara, CA, USA) following the manufacture’s protocol.

### 4.3. Transfection and Luciferase Assay

MCF-7, BT474, and MDA-MB-453 cells were plated into 96 well plate in full culture media. 24 h after transfection, pGL4.20-TFF1-Pro-WT (with wild type FOXA1 binding sites) and pGL4.20-TFF1-Pro-BS1, BS2, and BS1/2 (containing corresponding mutation in FOXA1 binding sites) were transfected into all cell lines with Lipofectamine 2000 (Invitrogen, Carlsbad, CA, USA) following the manufacture’s protocol. pRL-TK Renilla luciferase control reporter plasmid was co-transfected as the control for transfection efficiency. siRNA targeting FOXA1 (ON-TARGET J-010319-05-0005, Thermo Fisher Scientific, Waltham, MA, USA) or siControl Non-targeting (siNT) (SI03650318, Promega, Madison, WI, USA) were co-transfected with the pGL4.20-TFF1 reporter system with Lipofectamine 2000 (Invitrogen). Luciferase assay was directly carried out in the plate 48 h after transfection with the Dual-Glo Luciferase Reporter Assay System (E2920, Promega) following the manufacture’s protocol.

### 4.4. Chromatin Immunoprecipitation

Chromatin immunoprecipitation was performed as described previously [28]. In brief, first MCF-7 cells transfected with pGL4.20-TFF1 luciferase reporter were crosslinked with 1% formaldehyde for 10 min followed by quenching with 125 mM glycine. Then chromatin was sheared with sonication and incubated together with antibodies against FOXA1 (ab23738, Abcam, Cambridge, UK) or estrogen receptor alpha (sc-543, Santa Cruz Biotechnology, Dallas, TX, USA) respectively at 4 °C overnight. Immunoprecipitated DNA was purified with phenol-chloroform extraction followed by ethanol precipitation. FOXA1 and ER binding to the TFF1 luciferase reporter was detected by qPCR with a primer pair ranging across the restriction site used for cloning. The sequences of the qPCR primers are: CACCATGGAGAACAAGGTGA (forward) and AACAGTACCGGATTGCCAAG (reverse).

### 4.5. High Throughput Chemical Screening and Analysis

MCF-7, BT474, and MDA-MB-453 cells were plated into 10cm culture dish in complete media at 70% confluence. 24 h after plating, 15μg of pGL4.20-TFF1-Pro-WT was transfected with Lipofectamine 2000 following the manufacture’s protocol. 5 h after transfection, cells were washed with PBS and media was changed to clear DMEM medium supplemented with 5% stripped serum. After 2 h, transfected cells were plated into 384-well plate (781098, Greiner, Kremsmünster, Austria) in clear DMEM medium supplemented with 5% stripped serum. 24 h after transfection, cells were treated with selected chemical library at a final concentration of 10 μM (5 and 1 μM for the second screening). Three technical replicates were performed for each chemical. 24 h after the chemical treatment, luciferase assay was performed with Steady-Glo luciferase assay system (E2510, Promega) directly in 384-well plate following the manufacture’s protocol.

Positive chemicals were selected with Student’s *t*-test (*p*-value < 0.05, and with increase >50% or reduction >40% vs. vehicle). Heat map of the second screening was generated with Java TreeView Cluster 3.0 (https://sourceforge.net/projects/jtreeview/).

### 4.6. FOXA1 Phosphorylation Sites Prediction

Group-based Prediction System 3.0 (GPS 3.0) (http://gps.biocuckoo.org/) was used to predict potential phosphorylation sites in FOXA1 protein. Medium threshold was chosen for the analysis.

### 4.7. Immunoprecipitation

FOXA1 immunoprecipitation was performed with Pierce Crosslink IP kit (26147, Thermo Fisher Scientific) following the manufacture’s protocol. A total of 6 dishes (10 cm) of MCF-7 or BT474 cells cultured in full media were lyzed with Lysis/wash buffer. Cell lysate was loaded to columns containing protein A/G beads that cross-linked to anti-FOXA1 antibody (Abcam, ab23738). The incubation was kept at 4 °C for O/N followed by elusion with the Elusion buffer.

### 4.8. Protein Digestion

Total 50 µL of an IP protein extract with 200 µL of UA (Urea buffer) in the filter unit and centrifuge at 14,000× *g* for 15 min. Add 200 µL of UA to the filter unit and centrifuge at 14,000× *g* for 15 min. The flow-through was discarded. Next 100 µL IAA solution was added and mixed at 600 rpm in a thermo-mixer for 1 min and incubated without mixing for 20 min. Furthermore, the filter units were centrifuge at 14,000× *g* for 10 min. The filter unit was washed with adding 100 µL of UA and centrifuging at 14,000× *g* for 15 min. This step was repeated twice. Next the filter unit was equilibrated with 100 µL of ABC (ammonium bicarbonate) and centrifuge at 14,000× *g* for 10 min. This step as repeated twice. Finally, protein was digested by adding 40 µL ABC with trypsin (enzyme to protein ratio 1:50) and mix at 600 rpm in thermo-mixer for 1 min and Incubated the units at 37 °C for 18 h. Afterwards, the filter units was transfer to new collection tubes and centrifuged at 14,000× *g* for 10 min. One more time, 40 µL ABC was added and centrifuged at 14,000× *g* for 10 min to recover all digested peptides. Last filtrate was acidified with CF_3_COOH.

### 4.9. Desalting Digested Peptides

We used Oasis HLB cartridges (10 mg) from Waters (product no. 186000383, Oslo, Norway). Briefly, the following steps were followed: (1) condition the HLB cartridges with 1 mL 100% ACN (Acetonitrile); (2) equilibrate with 1.5 mL 2% ACN and 0.1% TFA buffer (wash solution); (3) load sample; (4) wash with 1 mL of wash solution; and (5) elute with 1 mL glycolic acid buffer (1 M glycolic acid, 5% TFA, 80% acetonitrile).

### 4.10. Data Processing and Analysis

Peptides were analyzed with Q-Exactive mass-spectrometry. Raw mass spectrometric data were analyzed in the MaxQuant tool and employed Andromeda for database search. The mass spectra were matched against the human Uniprot FASTA database. Enzyme specificity was set to trypsin, and the search included cysteine carbamidomethylation as a fixed modification and *N*-acetylation of protein, oxidation of methionine, and/ or phosphorylation of Ser, Thr, Tyr residue (STY) as variable modifications. Up to two missed cleavages were allowed for protease digestion, and peptides had to be fully tryptic.

## Supplementary Materials

Supplementary materials can be found at http://www.mdpi.com/1422-0067/19/12/4123/s1.
