# Supplementary material for: High Throughput Chemical Screening Reveals Multiple Regulatory Proteins on FOXA1 in Breast Cancer Cell Lines
Source: Int J Mol Sci. 2018 Dec 19;19(12):4123. doi: 10.3390/ijms19124123 (PMC6321185; doi:10.3390/ijms19124123)
Supplement: Supplementary file 1 [file ijms-19-04123-s001.zip › Supplementary Figures S1-S4.pdf]

Supplementary: High Throughput Chemical Screening Reveals Multiple Regulatory Proteins on FOXA1 in Breast Cancer Cell Lines

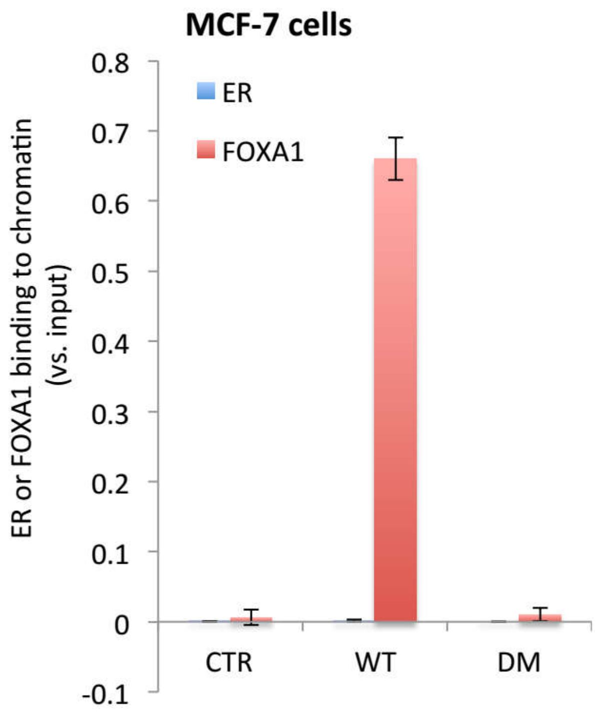

Figure S1. Analyzing the binding of FOXA1 or ER at the TTF1 promoter. MCF-7 cells were plated and then transfected with TFF1promoter-luciferase vector, empty vector (without TTF1 promoter) or with the TTF1-mutant-promoter vector (double mutant for forkheadmotifs). After transfection cells treated with hormone stripped media (media deprived of estrogen) as previously described [28]. After 24h of hormone deprivation, chromatin was cross-linked with formaldehyde and the isolated chromatin from each condition was splitted in two for ER or FOXA1 ChIP. We performed ChIP as previously described [12]. The binding of ER or FOXA1 at the TTF1 promoter was analyzed by realtime PCR of primers that specifically amplify the region across the TFF1 promoter and the N-terminus of luciferase coding region. The y-axis shows the binding of ER or FOXA1 normalized with input (isolated chromatin before ChIP) of regions amplifying control vector (CTR), TFF1 carrying wild type Forhkead motif (WT) or TTF1 carrying double mutant Forkhead motif (DM).

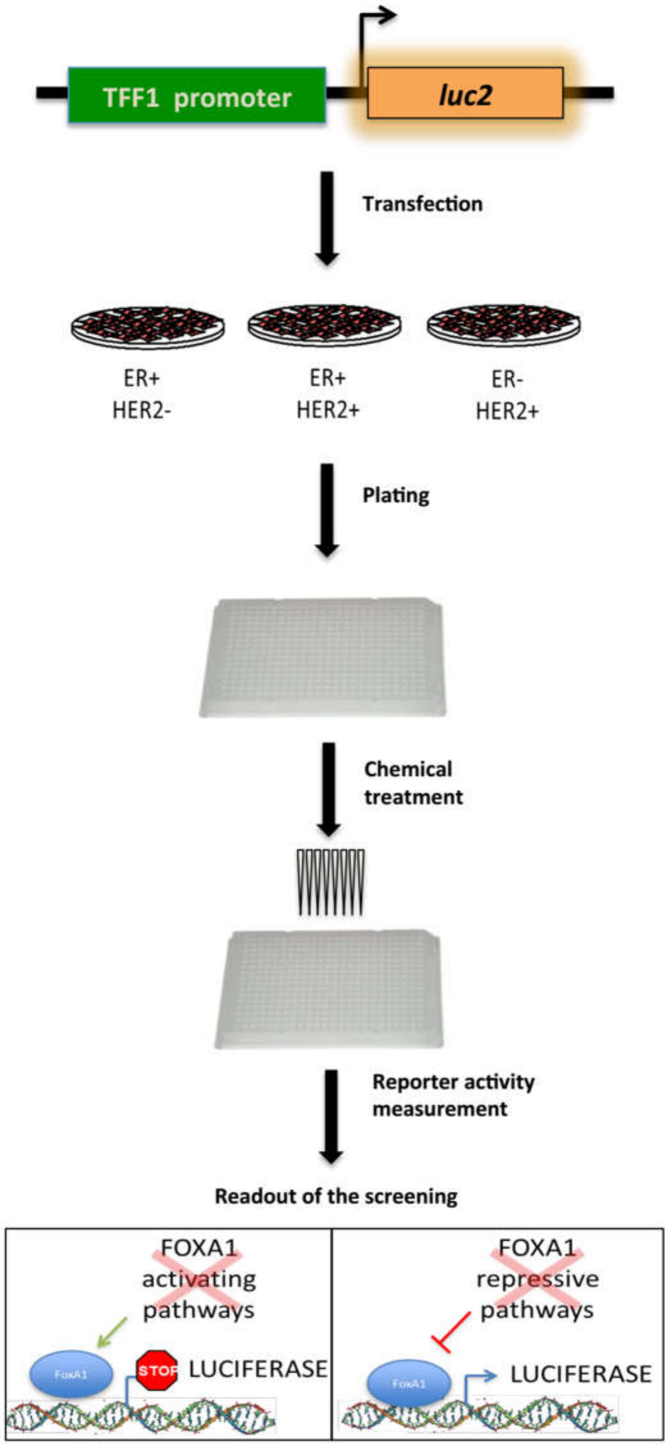

Figure S2. Pipeline of the high throughput chemical screening. pGL4.20-WT reporter plasmid was first transfected into MCF-7, BT474, and MDA-MB-453 cells in full culture media; 6 h after transfection cells were plated into 384 well plate in white DMEM with 5% hormone striped serum; 24 h after the plating, cells were treated with different chemicals from the library at concentration of 10 μM; 24 h after the start of the chemical treatment, luciferase assay was performed to measure the activity of the reporter.

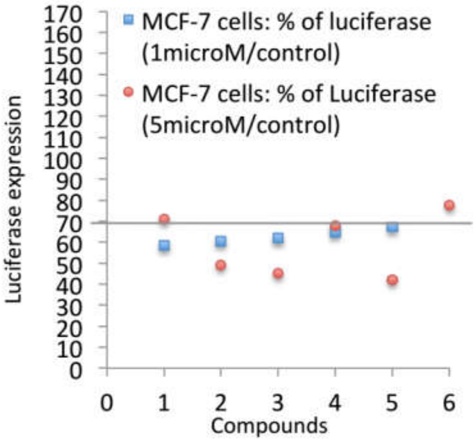

lane	compound	targets
1	Apicidin	HDAC inhibitor
2	Aurora A Inhibitor I	Aurora Kinase
3	Volasertib (BI 6727)	PLK
4	SNS-032 (BMS-387032)	CDK
5	Ro 31-8220	PKC
6	5-Iodotubercidin	ERK2, adenosine kinase, CK1, CK2,

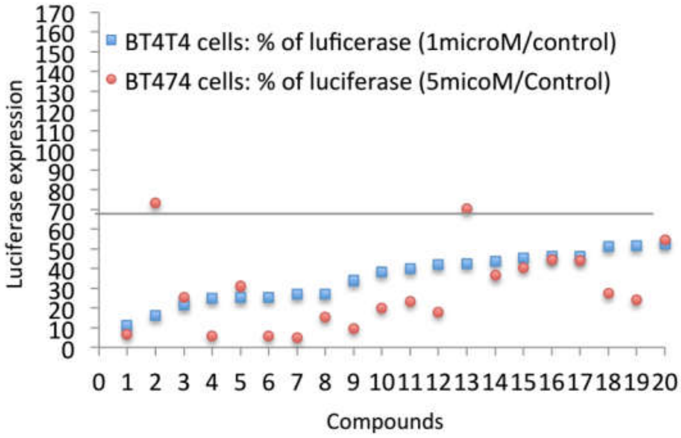

lane	Drug	target
1	WZ8040	EGFR
2	Flavopiridol	CDK
3	SNS-032 (BMS-387032)	CDK
4	Dasatinib	Bcr-Abl,c-Kit,Src
5	AT7519	CDK
6	Lapatinib	EGFR,HER2
7	Saracatinib (AZD0530)	Src,Bcr-Abl
8	Apicidin	HDAC inhibitor
9	Ro 31-8220	PKC
10	JNK-IN-8	JNK
11	BI 2536	PLK
12	Crenolanib (CP-868596)	PDGFR
13	Herceptin	EGFR,HER2
14	CYT387	JAK
15	BEZ235 (NVP-BEZ235, Dactolisib)	mTOR
16	PHA-793887	CDK
17	NVP-BSK805 2HCl	JAK
18	Cediranib (AZD2171)	VEGFR
19	Volasertib (BI 6727)	PLK
20	PF-00562271	FAK

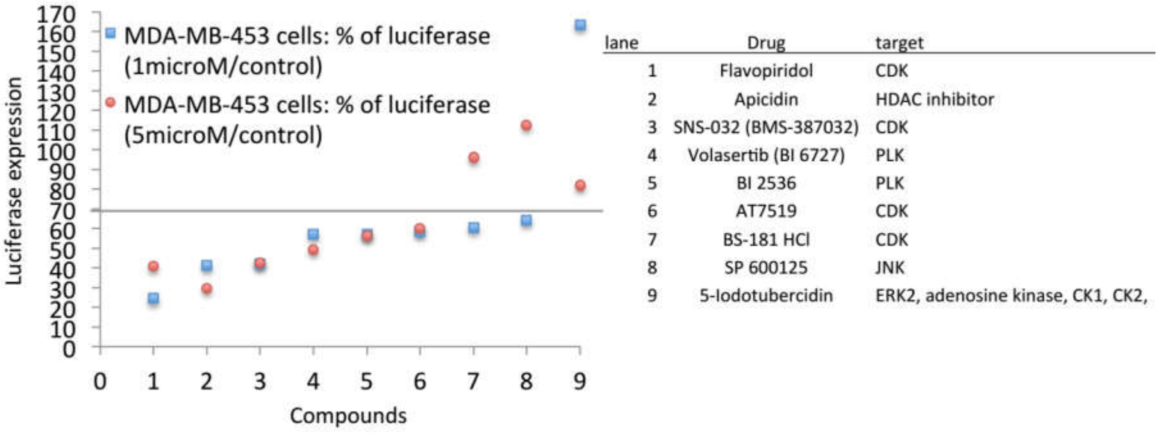

lane	Drug	target
1	Flavopiridol	CDK
2	Apicidin	HDAC inhibitor
3	SNS-032 (BMS-387032)	CDK
4	Volasertib (BI 6727)	PLK
5	BI 2536	PLK
6	AT7519	CDK
7	BS-181 HCl	CDK
8	SP 600125	JNK
9	5-Iodotubercidin	ERK2, adenosine kinase, CK1, CK2,

Figure S3. Complement of figure 3 and related with the second drug screening. The figure contains three plots and three tables. The three plots represent the luciferase expression (expressed as % of expression of each compound *vs.* control treated cells) of each of the cell lines analyzed (MCF-7, BT474 and MDA-MB-453). Each plot represents the % of inhibition of each compound at the concentration of 1 microM (blue) or 5 microM (red). The X axis indicates a number, which represents each of the compounds. The tables indicate the name of the compounds and their primary protein target.

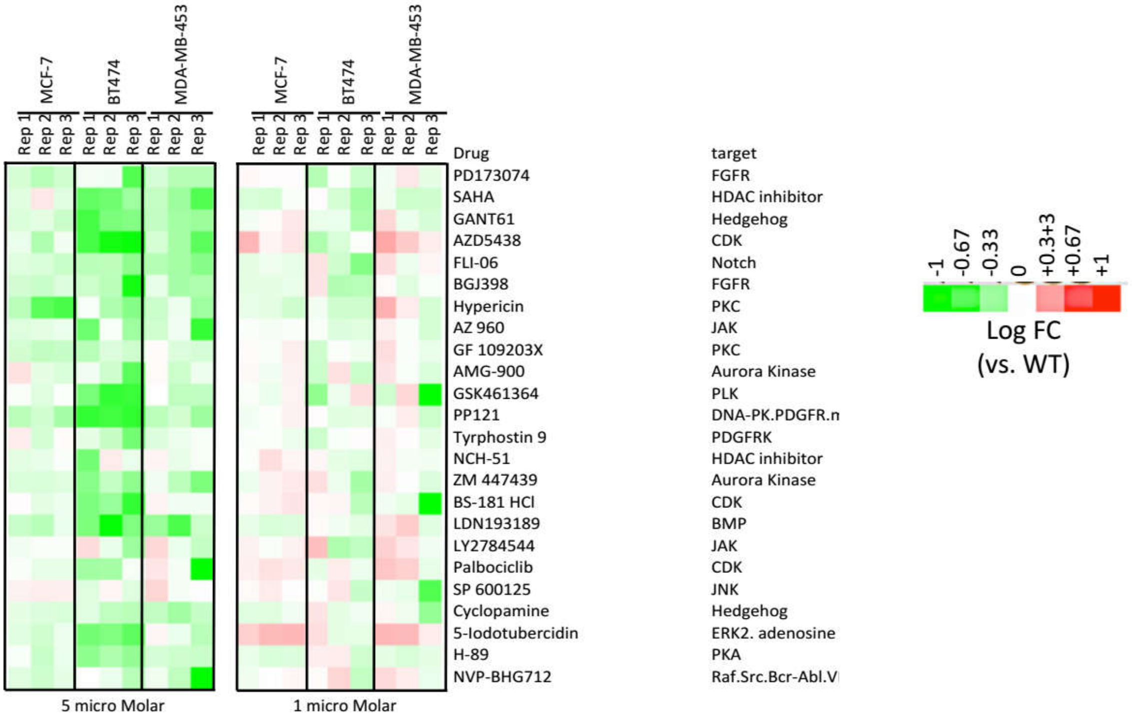

Figure S4. Validation of chemicals by the second screening. Heatmap showing the result of the compounds without a significant change in luciferase expression. 45 chemicals were used for the second chemical screening for 3 cell lines and 2 concentrations (5 and 1 microM). The heatmap illustrates the log2 fold change in luciferase expression (drug treatment vs. control) of cells treated with the 24 compounds without a significant p value.
