# Supplementary material for: High Throughput Chemical Screening Reveals Multiple Regulatory Proteins on FOXA1 in Breast Cancer Cell Lines
Source: Int J Mol Sci. 2018 Dec 19;19(12):4123. doi: 10.3390/ijms19124123 (PMC6321185; doi:10.3390/ijms19124123)
Supplement: Supplementary file 1 [file ijms-19-04123-s001.zip › Supplementary Table S2.pdf]

Table S2. List of significant FOXA1 interacting proteins identified by FOXA1 IP and MS. Separate lists for MCF-7 or BT474 cells are shown.

FOXA1 interacting proteins in BT474 cells

Protein names	Gene names	Unique peptides of rep1	Unique peptides of rep2	Unique peptides IgG
**Cyclin-dependent kinase 1**	CDK1	5	1	0
**YTH domain-containing family protein 1**	YTHDF3	1	1	0
**NADH dehydrogenase (ubiquinone) complex I**	NDUFAF6	1	1	0
**28S ribosomal protein S34**	MRPS34	1	3	0
**SH3 domain-binding glutamic acid-rich-like protein 3**	SH3BGRL3	2	2	0
**Required for meiotic nuclear division protein 1 homolog**	RMND1	1	1	0
**Vacuolar protein sorting-associated protein VTA1 homolog**	VTA1	1	1	0
**Transcription factor GATA-4**	GATA4	2	1	0
**Serine/arginine-rich splicing factor 7**	SRSF7	2	1	0
**Uncharacterized protein C11orf98**	C11orf98	1	1	0
**Bifunctional polynucleotide phosphatase/kinase**	PNKP	2	1	0
**Pleckstrin homology domain-containing family G member 3**	PLEKHG3	2	1	0
**39S ribosomal protein L28**	MRPL28	1	1	0
**von Willebrand factor A domain-containing protein 8**	VWA8	2	4	0
**Microtubule-associated proteins 1A/1B light chain 3B**	MAP1LC3B	2	1	0
**Phosphorylase b kinase regulatory subunit alpha**	PHKA1	1	1	0
**Heterochromatin protein 1-binding protein 3**	HP1BP3	1	1	0
**Coatomer subunit delta**	ARCN1	4	1	0
**Heterogeneous nuclear ribonucleoproteins C1/C2**	HNRNPC	5	3	0
**RNA-binding protein Musashi homolog 2**	MSI2	4	3	0
**Syntenin-1**	SDCBP	1	1	0
**Serine--tRNA ligase**	SARS2	1	2	0
**Zinc finger CCCH-type antiviral protein 1**	ZC3HAV1	3	1	0
**Integrator complex subunit 6**	INTS6	1	1	0
**DNA-directed RNA polymerases I**	POLR2H	1	1	0
**Peptidyl-prolyl cis-trans isomerase**	PPIH	4	1	0
**60S ribosomal protein L32**	RPL32	2	5	0
**Transcription factor BTF3**	BTF3	1	1	0
**Nucleotide exchange factor SIL1**	SIL1	1	2	0
**Dynactin subunit 6**	DCTN6	1	1	0
**Cytochrome c oxidase subunit 7A-related protein**	COX7A2L	1	1	0
**Protein FAM98A**	FAM98A	1	1	0
**60S ribosomal protein L8**	RPL8	2	2	0
**NADH dehydrogenase [ubiquinone] iron-sulfur protein 8**	NDUFS8	1	1	0
**Protein transport protein Sec16A**	SEC16A	4	2	0
**DNA repair protein XRCC1**	XRCC1	4	3	0
**Ubiquitin-conjugating enzyme E2 N**	UBE2N	2	1	0
**Charged multivesicular body protein 1a**	CHMP1A	3	2	0

**Vacuolar protein sorting-associated protein 29**	VPS29	2	1	0
**Nesprin-3**	SYNE3	1	1	0
**60S ribosomal protein L3**	RPL3	3	3	0
**Dolichol-phosphate mannosyltransferase subunit 1**	DPM1	7	6	0
**Receptor-type tyrosine-protein phosphatase F**	PTPRF	1	2	0
**Mitochondrial fission regulator 1**	MTFR1	1	1	0
**SRSF protein kinase 1**	SRPK1	2	1	0
**26S proteasome non-ATPase regulatory subunit 9**	PSMD9	3	3	0
	ALOXE3	1	1	0
**AP-2 complex subunit sigma**	AP2S1	2	2	0
**PDZ and LIM domain protein 1**	PDLIM1	1	1	0
**U6 snRNA-associated Sm-like protein LSm1**	LSM1	1	2	0
**NADH dehydrogenase [ubiquinone] 1 alpha subcomplex subunit 2**	NDUFA2	1	1	0
**Probable 18S rRNA (guanine-N(7))-methyltransferase**	WBSCR22	1	1	0
**LanC-like protein 1**	LANCL1	4	2	0
**Protein SCO2 homolog**	SCO2	2	1	0
**Tripartite motif-containing protein 3**	TRIM3	2	2	0
**Vacuolar protein sorting-associated protein 26A**	VPS26A	1	1	0
**Serine/arginine-rich splicing factor 10**	SRSF10	1	1	0
**Heat shock factor-binding protein 1**	HSBP1	2	1	0
**Barrier-to-autointegration factor**	BANF1	3	2	0
**Interferon-inducible double-stranded RNA-dependent protein kinase activator A**	PRKRA	5	5	0
**NADH dehydrogenase [ubiquinone] 1 beta subcomplex subunit 4**	NDUFB4	2	2	0
**Probable tRNA pseudouridine synthase 2**	TRUB2	2	1	0
**2,3-cyclic-nucleotide 3-phosphodiesterase**	CNP	1	1	0
**Ubiquitin-like protein 4A**	UBL4A	1	1	0
**Heterogeneous nuclear ribonucleoprotein L**	HNRNPL	2	2	0
**High mobility group protein HMG-I/HMG-Y**	HMGA1	1	1	0
**60S ribosomal protein L7**	RPL7	4	5	0
**Heterogeneous nuclear ribonucleoprotein H3**	HNRNPH3	1	1	0
**Replication protein A 14 kDa subunit**	RPA3	2	1	0
**Replication factor C subunit 4**	RFC4	5	4	0
**60S ribosomal protein L13a**	RPL13A	2	2	0
**60S ribosomal protein L28**	RPL28	6	5	0
**60S ribosomal protein L34**	RPL34	1	2	0
**Hepatocyte nuclear factor 3-alpha**	FOXA1	9	8	0
**60S ribosomal protein L27**	RPL27	4	4	0
**DDB1- and CUL4-associated factor 7**	DCAF7	6	2	0
**WD repeat-containing protein 5**	WDR5	5	6	0
**40S ribosomal protein S8**	RPS8	5	5	0
**DNA-directed RNA polymerases I**	POLR2L	2	2	0
**Putative 60S ribosomal protein L39-like 5**	RPL39P5	1	1	0

**Growth factor receptor-bound protein 2**	GRB2	3	1	0
**Transformer-2 protein homolog beta**	TRA2B	2	1	0
**28S ribosomal protein S6**	MRPS6	2	2	0
**Transcription factor A**	TFAM	5	3	0
**Serine/arginine-rich splicing factor 9**	SRSF9	3	3	0
**SNW domain-containing protein 1**	SNW1	2	1	0
**ELAV-like protein 1**	ELAVL1	7	1	0
**Myeloid leukemia factor 2**	MLF2	2	1	0
**Mitochondrial import receptor subunit TOM34**	TOMM34	4	4	0
**UDP-N-acetylhexosamine pyrophosphorylase**	UAP1	1	1	0
**WD40 repeat-containing protein SMU1**	SMU1	4	3	0
**PDZ domain-containing protein 11**	PDZD11	2	1	0
**Cancer-related nucleoside-triphosphatase**	NTPCR	1	2	0
**Pleckstrin homology domain-containing family A member 6**	PLEKHA6	2	1	0
**Ankyrin repeat domain-containing protein 22**	ANKRD22	1	1	0
**Tetratricopeptide repeat protein 19**	TTC19	3	3	0
**39S ribosomal protein L54**	MRPL54	1	1	0
**2-oxoglutarate and iron-dependent oxygenase domain-containing protein 3**	OGFOD3	1	1	0
**Dehydrogenase/reductase SDR family member 13**	DHRS13	1	1	0
**WD repeat-containing protein 82**	WDR82	2	2	0
**Charged multivesicular body protein 1b**	CHMP1B	2	2	0
**DnaJ homolog subfamily C member 10**	DNAJC10	6	7	0
**Activating signal cointegrator 1 complex subunit 1**	ASCC1	3	1	0
**ATPase family AAA domain-containing protein 1**	ATAD1	2	1	0
**UDP-glucuronic acid decarboxylase 1**	UXS1	1	1	0
**LEM domain-containing protein 2**	LEMD2	4	3	0
**Deleted in autism protein 1**	C3orf58	2	1	0
**Alpha/beta hydrolase domain-containing protein 11**	ABHD11	6	5	0
**Phosphatidylglycerophosphatase and protein-tyrosine phosphatase 1**	PTPMT1	4	2	0
**Dimethyladenosine transferase 1**	TFB1M	1	1	0
**Ataxin-2-like protein**	ATXN2L	3	2	0
**5-methylcytosine rRNA methyltransferase NSUN4**	NSUN4	2	2	0
**Coiled-coil domain-containing protein 124**	CCDC124	1	1	0
**Pentatricopeptide repeat domain-containing protein 3**	PTCD3	2	1	0
**Bcl10-interacting CARD protein**	C9orf89	2	5	0
**Zinc finger protein 385A**	ZNF385A	2	2	0
**Protein S100-A13**	S100A13	2	1	0
**Evolutionarily conserved signaling intermediate in Toll pathway**	ECSIT	4	1	0
**Dehydrogenase/reductase SDR family member 4**	DHRS4	2	2	0
**HIG1 domain family member 2A**	HIGD2A	1	1	0
**Uncharacterized protein KIAA1671**	KIAA1671	3	5	0
**Phosducin-like protein 3**	PDCL3	3	1	0

**LYR motif-containing protein 4**	LYRM4	2	2	0
**PDZ and LIM domain protein 7**	PDLIM7	2	2	0
**Protein FAM207A**	FAM207A	2	2	0
**H/ACA ribonucleoprotein complex subunit 1**	GAR1	2	1	0
**NADH dehydrogenase [ubiquinone] 1 alpha subcomplex assembly factor 4**	NDUFAF4	4	3	0
**Cytochrome b-c1 complex subunit 9**	UQCR10	2	2	0
**NADH dehydrogenase [ubiquinone] 1 alpha subcomplex subunit 12**	NDUFA12	4	1	0
**Dynactin subunit 4**	DCTN4	2	2	0
**mRNA turnover protein 4 homolog**	MRTO4	4	2	0
**Protein kinase C and casein kinase substrate in neurons protein 2**	PACSIN2	2	1	0
**Probable dimethyladenosine transferase**	DIMT1	1	2	0
**Leydig cell tumor 10 kDa protein homolog**	C19orf53	2	3	0
**39S ribosomal protein L11**	MRPL11	6	5	0
**Peptidyl-prolyl cis-trans isomerase-like 1**	PPIL1	2	4	0
**Mitochondrial fission 1 protein**	FIS1	1	1	0
**60S ribosomal protein L36**	RPL36	4	3	0
**U6 snRNA-associated Sm-like protein LSm4**	LSM4	1	1	0

FOXA1 interacting proteins in MCF7 cells

**Protein namesProtein names**	**Gene names**	**Unique peptides of rep1**	**Unique peptides of rep2**	**Unique peptides IgG**
**28S ribosomal protein S34**	MRPS34	1	1	0
**Required for meiotic nuclear division protein 1 homolog**	RMND1	1	2	0
**DDRGK domain-containing protein 1**	DDRGK1	1	1	0
**Coronin**	PAM16	2	1	0
**40S ribosomal protein S15**	RPS15	4	3	0
**Dehydrogenase/reductase SDR family member 7B**	DHRS7B	2	2	0
**Vacuolar protein sorting-associated protein 37C**	VPS37C	2	1	0
**Protein PBDC1**	PBDC1	1	1	0
**Non-histone chromosomal protein HMG-14**	HMGN1	1	1	0
**Tuftelin**	TUFT1	1	1	0
**Small nuclear ribonucleoprotein G**	SNRPG	1	1	0
**NHP2-like protein 1**	NHP2L1	1	1	0
**Lymphocyte function-associated antigen 3**	CD58	1	1	0
**Heterogeneous nuclear ribonucleoproteins C1/C2**	HNRNPC	2	2	0
**RNA-binding protein Musashi homolog 2**	MSI2	3	2	0
**Eukaryotic translation initiation factor 4E type 2**	EIF4E2	1	1	0
**RNA-binding protein with multiple splicing**	RBPMS	3	2	0
**Charged multivesicular body protein 2b**	CHMP2B	1	1	0
**DNA-directed RNA polymerases I**	POLR2H	1	1	0

**Uncharacterized protein C7orf50**	C7orf50	2	5	0
**CGG triplet repeat-binding protein 1**	CGGBP1	1	1	0
**60S ribosomal protein L22-like 1**	RPL22L1	1	2	0
**Transmembrane protein 33**	TMEM33	1	1	0
**Uncharacterized protein C1orf198**	C1orf198	1	1	0
**Centrin-3**	CETN3	1	1	0
**Transforming acidic coiled-coil-containing protein 2**	TACC2	15	19	0
**60S ribosomal protein L14**	RPL14	3	3	0
**RNA-binding motif**	RBMS1	2	1	0
**Selenoprotein S**	VIMP	1	1	0
**Midkine**	MDK	1	1	0
**G-rich sequence factor 1**	GRSF1	1	1	0
**DNA repair protein XRCC1**	XRCC1	2	2	0
**Charged multivesicular body protein 1a**	CHMP1A	3	2	0
**SWI/SNF complex subunit SMARCC2**	SMARCC2	1	1	0
**60S ribosomal protein L3**	RPL3	2	1	0
**Dolichol-phosphate mannosyltransferase subunit 1**	DPM1	1	2	0
**Histone deacetylase**	HDAC7	1	1	0
**Rab-3A-interacting protein**	RAB3IP	1	1	0
**Synaptosomal-associated protein**	SNAP23	2	1	0
**Transcription factor 25**	TCF25	1	1	0
**26S proteasome non-ATPase regulatory subunit 9**	PSMD9	2	1	0
**Mitochondrial inner membrane protein OXA1L**	OXA1L	1	1	0
**60S ribosomal protein L36a**	RPL36A	3	2	0
**Transmembrane and coiled-coil domain-containing protein 1**	TMCO1	2	1	0
**Protein FAM32A**	FAM32A	1	1	0
**DAZ-associated protein 1**	DAZAP1	2	1	0
**PIH1 domain-containing protein 1**	PIH1D1	1	1	0
**Centrosomal protein of 290 kDa**	CEP290	1	1	0
**Mitochondrial ribonuclease P protein 3**	KIAA0391	5	4	0
**Protein regulator of cytokinesis 1**	PRC1	1	1	0
**PC4 and SFRS1-interacting protein**	PSIP1	1	2	0
**Interferon-inducible double-stranded RNA-dependent protein kinase activator A**	PRKRA	7	5	0
**Zinc finger protein-like 1**	ZFPL1	1	1	0
**NADH dehydrogenase [ubiquinone] 1 beta subcomplex subunit 4**	NDUFB4	1	1	0
**NADH dehydrogenase [ubiquinone] 1 alpha subcomplex subunit 7**	NDUFA7	1	1	0
**CD2 antigen cytoplasmic tail-binding protein 2**	CD2BP2	1	1	0
**BAG family molecular chaperone regulator 2**	BAG2	1	2	0
**Cytochrome b-c1 complex subunit 6**	UQCRH	4	5	0
**Chromosome transmission fidelity protein 8 homolog isoform 2**	CHTF8	4	3	0
**Myosin-6**	MYH6	1	0	0
**Heterogeneous nuclear ribonucleoprotein L**	HNRNPL	1	1	0

**High mobility group protein HMG-I/HMG-Y**	HMGA1	2	1	0
**60S ribosomal protein L7**	RPL7	3	5	0
**ATP synthase-coupling factor 6**	ATP5J	3	2	0
**RNA-binding motif protein**	RBMX	3	4	0
**Replication factor C subunit 5**	RFC5	4	5	0
**60S ribosomal protein L21**	RPL21	4	4	0
**IST1 homolog**	IST1	2	2	0
**Atrophin-1**	ATN1	1	1	0
**Hepatocyte nuclear factor 3-alpha**	FOXA1	9	10	0
**DNA-directed RNA polymerases I**	POLR2L	1	1	0
**40S ribosomal protein S21**	RPS21	2	1	0
**mRNA export factor**	RAE1	1	1	0
**28S ribosomal protein S10**	MRPS10	1	1	0
**28S ribosomal protein S35**	MRPS35	2	2	0
**Serine/arginine-rich splicing factor 3**	SRSF3	5	3	0
**RNA-binding protein 3**	RBM3	2	2	0
**Transcription factor A**	TFAM	1	1	0
**Lactadherin**	MFGE8	1	2	0
**Nuclear cap-binding protein subunit 1**	NCBP1	2	4	0
**Serine/arginine-rich splicing factor 9**	SRSF9	2	2	0
**SNW domain-containing protein 1**	SNW1	2	1	0
**E3 ubiquitin/ISG15 ligase TRIM25**	TRIM25	1	1	0
**ELAV-like protein 1**	ELAVL1	3	2	0
**WD40 repeat-containing protein SMU1**	SMU1	1	1	0
**Mitochondrial import inner membrane translocase subunit TIM50**	TIMM50	2	2	0
**39S ribosomal protein L54**	MRPL54	1	1	0
**WD repeat-containing protein 82**	WDR82	2	1	0
**Heparan sulfate 2-O-sulfotransferase 1**	HS2ST1	1	1	0
**PHD finger-like domain-containing protein 5A**	PHF5A	1	1	0
**Protein MON2 homolog**	MON2	4	6	0
**Testis-specific Y-encoded-like protein 5**	TSPYL5	1	1	0
**MICAL-like protein 2**	MICALL2	3	4	0
**Uncharacterized protein KIAA1958**	KIAA1958	1	1	0
**LEM domain-containing protein 2**	LEMD2	2	2	0
**DnaJ homolog subfamily C member 9**	DNAJC9	3	4	0
**Zinc finger protein 385A**	ZNF385A	1	2	0
**Plakophilin-2**	PKP2	1	1	0
**Melanophilin**	MLPH	1	1	0
**5-3 exoribonuclease 2**	XRN2	2	1	0
**Charged multivesicular body protein 4b**	CHMP4B	1	1	0
**Vacuolar protein sorting-associated protein 37B**	VPS37B	1	1	0
**Protein FAM207A**	FAM207A	3	4	0
**NADH dehydrogenase [ubiquinone] 1 alpha subcomplex subunit 12**	NDUFA12	3	2	0

**SH3 domain-binding glutamic acid-rich-like protein 2**	SH3BGRL2	2	1	0
**Heat shock protein beta-8**	HSPB8	1	2	0
**mRNA turnover protein 4 homolog**	MRTO4	4	4	0
**Leydig cell tumor 10 kDa protein homolog**	C19orf53	1	1	0
**28S ribosomal protein S28**	MRPS28	1	1	0
**Thyroid hormone receptor-associated protein 3**	THRAP3	5	6	0
**28S ribosomal protein S2**	MRPS2	1	1	0
**Splicing factor 3B subunit 6**	SF3B6	1	1	0
**39S ribosomal protein L11**	MRPL11	5	3	0
**Peptidyl-prolyl cis-trans isomerase-like 1**	PPIL1	2	2	0
**Mitochondrial import inner membrane translocase subunit Tim13**	TIMM13	1	1	0
**DnaJ homolog subfamily C member 15**	DNAJC15	1	1	0
